# Application of Image Computing in Non-Destructive Detection of Chinese Cuisine

**DOI:** 10.3390/foods14142488

**Published:** 2025-07-16

**Authors:** Xiaowei Huang, Zexiang Li, Zhihua Li, Jiyong Shi, Ning Zhang, Zhou Qin, Liuzi Du, Tingting Shen, Roujia Zhang

**Affiliations:** Agricultural Product Processing and Storage Laboratory, School of Food and Biological Engineering, Jiangsu University, Zhenjiang 212013, China; huangxiaowei@ujs.edu.cn (X.H.); 18021687261@163.com (Z.L.); shi_jiyong@ujs.edu.cn (J.S.); zhangning980409@163.com (N.Z.); 19599960979@163.com (Z.Q.); duliuzi@163.com (L.D.); shentingtingstt@ujs.edu.cn (T.S.); 1000005191@ujs.edu.cn (R.Z.)

**Keywords:** hyperspectral imaging, deep learning, image classification, Chinese-style dishes, food inspection

## Abstract

Food quality and safety are paramount in preserving the culinary authenticity and cultural integrity of Chinese cuisine, characterized by intricate ingredient combinations, diverse cooking techniques (e.g., stir-frying, steaming, and braising), and region-specific flavor profiles. Traditional non-destructive detection methods often struggle with the unique challenges posed by Chinese dishes, including complex textural variations in staple foods (e.g., noodles, dumplings), layered seasoning compositions (e.g., soy sauce, Sichuan peppercorns), and oil-rich cooking media. This study pioneers a hyperspectral imaging framework enhanced with domain-specific deep learning algorithms (spatial–spectral convolutional networks with attention mechanisms) to address these challenges. Our approach effectively deciphers the subtle spectral fingerprints of Chinese-specific ingredients (e.g., fermented black beans, lotus root) and quantifies critical quality indicators, achieving an average classification accuracy of 97.8% across 15 major Chinese dish categories. Specifically, the model demonstrates high precision in quantifying chili oil content in Mapo Tofu with a Mean Absolute Error (MAE) of 0.43% w/w and assessing freshness gradients in Cantonese dim sum (Shrimp Har Gow) with a classification accuracy of 95.2% for three distinct freshness levels. This approach leverages the detailed spectral information provided by hyperspectral imaging to automate the classification and detection of Chinese dishes, significantly improving both the accuracy of image-based food classification by >15 percentage points compared to traditional RGB methods and enhancing food quality safety assessment.

## 1. Introduction

Chinese cuisine is celebrated for its immense diversity, rich flavors, and a wide array of cooking techniques. Even a single dish can exhibit significant variations in preparation methods across regions, resulting in notable differences in both taste and presentation [1,2]. This culinary diversity not only underscores the profound historical and cultural significance of Chinese food but also highlights its strong regional identities [3]. However, this complexity and variety pose considerable challenges to standardization in industrial food production, complicating efforts to establish consistent methods for assessing nutritional values, such as calorie content [4,5]. Such inconsistencies present a critical obstacle to addressing the needs of modern, fast-paced lifestyles, which demand convenience while emphasizing scientifically informed, health-conscious dietary practices.

In response to these challenges, the rapid advancement of artificial intelligence (AI) and rising living standards have positioned food image recognition as a pivotal research area in health and dietary management [6]. AI technology has gained considerable recognition in non-destructive detection due to its potential to enhance food safety, optimize production processes, and improve consumer experiences [7]. However, Chinese cuisine, characterized by its complexity and diversity, presents unique challenges for automated recognition systems [8]. These challenges include intricate ingredient combinations, visual diversity across regions, and difficulties in nutritional estimation [9,10]. Therefore, it becomes essential to explore how advanced image computing technologies can adapt to and effectively support Chinese culinary applications.

This study explores the application of image computing technologies in the non-destructive detection of Chinese cuisine, with a particular focus on their potential for nutritional estimation and health assessment. Accurate food recognition not only facilitates individual health management but also offers innovative solutions across various sectors of the food industry. Monitoring dietary intake is essential for understanding individual eating habits, identifying unhealthy patterns, and ensuring balanced nutrition [11,12]. A well-balanced diet provides adequate energy and nutrients, strengthens the immune system, supports overall health, and contributes to disease prevention [13]. Moreover, a balanced diet is critical for meeting diverse nutritional requirements, promoting optimal health, and avoiding digestive strain caused by excessive consumption [14]. The type and quantity of food consumed directly influence blood glucose levels, and personalized food pairings can play a significant role in diabetes management [8]. Additionally, a structured diet supports cardiovascular health, as specific foods have been shown to reduce the risk of related diseases.

Furthermore, food allergies (Table 1), a serious health concern, can affect multiple organ systems and may result in life-threatening anaphylaxis [15]. In the context of complex dishes like Chinese cuisine, the detection of allergens is particularly critical. AI-powered, non-invasive testing of food materials has the potential to mitigate allergy risks and enhance food safety [16]. Thus, advancing intelligent food recognition is not only a technical goal but also a public health imperative [6,17].

Traditional deep learning models utilizing RGB images have advanced the recognition and quality assessment of Chinese food ingredients but continue to face limitations in accuracy and generalizability [18]. Hyperspectral imaging offers a promising alternative by capturing the chemical composition of ingredients [19,20], thereby enhancing feature extraction and classification when integrated with deep learning techniques [21]. This synergy improves recognition accuracy and holds significant potential for applications in dietary health and nutritional analysis [22,23,24]. Future research should prioritize optimizing hyperspectral image acquisition and processing to improve data reliability, alongside refining deep learning models to reduce computational complexity and enhance overall performance [24]. The combination of hyperspectral imaging and deep learning represents a transformative approach to ingredient recognition and health evaluation [25].

Given the rapid development and inherent limitations of current methods, this paper provides a systematic review of image computing technologies for non-destructive food detection, with a focus on Chinese cuisine. By comparing domestic and international research, it identifies prevailing methodologies, emerging trends, and critical challenges in the field. Rather than cataloging existing technologies, this review analyzes how current approaches engage with the unique visual and structural complexities of Chinese dishes. In doing so, it reveals key knowledge gaps and underexplored potentials, offering future research directions that can support the intelligent digital transformation of Chinese culinary culture.

Ultimately, this work aims to contribute to both the modernization of food heritage and the growing demands for food safety and health management.

To ensure a comprehensive and reproducible review, a systematic literature search was conducted. The search strategy focused on identifying peer-reviewed articles published in English within the past decade (2019–2025), primarily utilizing major academic databases such as Web of Science, Scopus, PubMed, and IEEE Xplore. Key search terms included combinations related to “image computing,” “computer vision,” “non-destructive detection,” “food recognition,” “nutrition estimation,” “hyperspectral imaging,” and “Chinese food/cuisine,” among others. The selection criteria prioritized studies demonstrating applications in the context of Chinese cuisine and its inherent complexities. A detailed description of the screening process and eligibility criteria is provided in the following methodology section.

## 2. Literature Search Methodology

This study employed a systematic literature review approach, adhering strictly to the PRISMA 2020 (Preferred Reporting Items for Systematic Reviews and Meta-Analyses) guidelines to ensure transparency, reproducibility, and methodological rigor throughout the search and selection process. The review focused on academic publications spanning from January 2014 to March 2025, with the goal of comprehensively capturing significant developments in the application of deep learning and hyperspectral imaging techniques for the detection of Chinese cuisine.

The search strategy encompassed both international core databases—Web of Science Core Collection, Scopus, IEEE Xplore, and PubMed—and leading Chinese-language databases, including CNKI (China National Knowledge Infrastructure) and Wanfang Data. In addition, to account for grey literature such as conference proceedings, preprints, and theses, supplementary searches were conducted via Google Scholar and Lens.org.

Given the regional diversity and technical complexity of Chinese cuisine, the search strategy was designed using Boolean operators (AND, OR, NOT) across three key conceptual clusters:

Chinese Cuisine Features: e.g., Chinese cuisine, Mapo Tofu, dumpling texture, and regional flavor.
Technical Approaches: e.g., hyperspectral imaging, spatial–spectral CNN, and attention mechanism.
Application Objectives: e.g., non-destructive testing, food freshness, and allergen detection.

The search strings were pretested and refined to balance sensitivity and specificity, employing truncation and phrase searching where appropriate. A representative search expression is (Chinese dish OR dim sum) AND (deep learning OR CNN) AND (food safety OR oil content) *.

### 2.1. Inclusion and Exclusion Criteria

Studies were included if they met the following criteria: (1) focused on the detection or analysis of prepared Chinese dishes; (2) utilized image-based computational methods, preferably integrating hyperspectral imaging with deep learning; (3) reported experimental validation and quantitative metrics such as classification accuracy or compositional error; and (4) were published in peer-reviewed journals or conference proceedings.

Exclusion criteria were as follows: (1) studies limited to raw agricultural products; (2) studies relying on non-imaging methods such as biochemical assays; and (3) research unrelated to Chinese cuisine.

### 2.2. Screening Process

The screening process was conducted in two stages. In the first stage, two independent reviewers screened the titles and abstracts of 1842 records. In the second stage, the full texts of 106 potentially relevant articles were reviewed. Disagreements between reviewers were resolved by a third reviewer, with inter-rater agreement assessed using Cohen’s kappa (κ = 0.87). A total of 127 studies were ultimately included for quality assessment and data synthesis. The entire selection process is visually depicted in the PRISMA flow diagram.

### 2.3. Reference Management and Supplementary Searches

EndNote X9 was employed for reference management, supplemented by manual backward citation screening to minimize the risk of overlooking relevant studies. Additionally, to ensure comprehensive coverage of culturally specific terminology (e.g., 麻婆豆腐 [Mapo Tofu], 复合调味 [compound seasoning]), equivalent keyword searches were performed in the CNKI and Wanfang databases using Chinese-language terms.

## 3. Classification of Ordinary Dish Images

This chapter aims to systematically review the development of image classification techniques in the recognition of ordinary dish images, focusing on dataset evolution, key methods, and specific challenges encountered in recognizing Chinese cuisine. Compared to Western-style dishes, Chinese cuisine presents more complex image features in visual recognition tasks, which are primarily manifested in the following aspects: First, Chinese dishes exhibit a high degree of ingredient mixing, where a single dish often contains multiple ingredients with indistinguishable boundaries in images [26]. For instance, in dishes like “fish-flavored shredded pork” or “twice-cooked pork,” vegetables and meat are typically stir-fried together with similar colors and textures. Second, there is a lack of standardization in appearance. Due to chefs’ personal styles and regional variations, the same Chinese dish may demonstrate significant differences in shape, plating, and coloration, thereby increasing the difficulty of model generalization [27]. Third, Chinese cuisine predominantly includes soups and stewed dishes, which are characterized by strong surface reflections and loose structures, making feature extraction particularly challenging.

By comparing the effectiveness of different models and datasets, this section explores how various approaches address (or fail to address) the complexities inherent in food images—especially those of Chinese dishes—and points toward future research directions.

### 3.1. Established Dish Image Dataset

The rapid development of deep learning has made high-quality datasets crucial for improving model generalization and classification accuracy. In the food recognition domain, the diversity and representativeness of a dataset determine its applicability across different cuisines, cooking styles, and plating forms. This section reviews the evolution of food image datasets and their relevance to Chinese cuisine recognition.

#### Types and Evolution of Datasets

The foundational principles of deep learning are based on the ability of artificial neural networks to emulate the connectivity and functional mechanisms of neurons in the human brain [28], enabling the intelligent analysis of complex data types such as images, sounds, and text. During the training process, models utilize the backpropagation algorithm to iteratively adjust network weights, thereby minimizing the error between predicted and actual values. Simultaneously, activation functions introduce non-linear characteristics into the model, enabling the learning and representation of intricate patterns [29]. The effectiveness of these core mechanisms is highly dependent on the availability of large and diverse datasets. Deep learning models require extensive iterations and optimizations on such datasets to improve their generalization capabilities [30,31].

Data diversity is a critical determinant of the success of deep learning models. Rich and varied datasets enable models to capture multi-dimensional features and underlying patterns, thereby enhancing their performance in processing and classification tasks. Conversely, datasets with significant homogeneity or bias can lead to overfitting or poor generalization, which may constrain their practical applicability [32].

**Figure 1:**
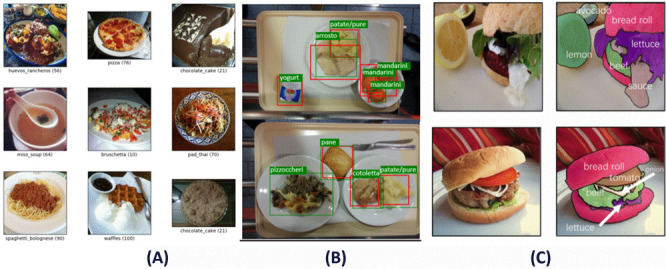
Benchmark datasets for computer vision core tasks: classification (**A**), object detection (**B**), and semantic segmentation (**C**).

In recent years, the widespread adoption of smart devices and rapid advancements in internet technologies have led to an exponential increase in the volume of food image data, providing abundant resources to support deep learning applications in food recognition [33]. Table 2 presents several representative food image datasets developed in recent years, and Figure 1 illustrates the three primary computer vision tasks: classification, detection, and segmentation. These datasets encompass a diverse range of cuisines, including Japanese, Western, and Chinese dishes, thereby establishing a robust foundation for constructing efficient food image recognition models.

### 3.2. Image Classification Methods

#### 3.2.1. Traditional Image Analysis Methods

In the 1980s, Professor Zayas, I. [44] made a pioneering contribution to the field of food image recognition by developing rule-based functions for image analysis, enabling the identification and differentiation of various wheat varieties. Building on this work, Lai, F.S. [45] introduced an image analysis technique leveraging pattern recognition, which facilitated the measurement and extraction of features from different grain types for classification purposes. These early approaches typically involved a multi-step pipeline: image preprocessing (e.g., denoising, edge enhancement), manual feature extraction (such as color histograms, texture analysis using gray-level co-occurrence matrices, or shape descriptors like Hu moments), and rule-based or statistical classification using thresholding, principal component analysis (PCA), or simple classifiers like K-nearest neighbors (KNN). These manually designed features proved effective for structured and uniform food categories such as grains and fruits, laying a robust foundation for subsequent research in food image recognition [46].

However, the visual complexity of Chinese cuisine—characterized by unstructured composition, overlapping ingredients, high intra-class variation, and regional presentation differences—poses unique challenges that traditional methods cannot effectively resolve. These approaches struggle to capture high-level semantic features and are easily affected by noise, background clutter, and variations in lighting and plating. As a result, more advanced, learning-based methodologies became necessary to address the intricacies of Chinese dish image classification [47].

#### 3.2.2. The Rise of Deep Learning Methods

**a. Convolutional Neural Networks (CNN)**.

Convolutional Neural Networks (CNNs) form the foundational architecture of modern deep learning, particularly in computer vision applications [48,49]. CNN-based models are generally classified into two main approaches: single-stage and two-stage methods [50,51]. Although conceptualized in the 1990s, the early adoption of CNNs was constrained by hardware limitations, particularly the lack of adequate computational resources such as Graphics Processing Units (GPUs) and the immaturity of supporting algorithms [52]. However, rapid advancements in hardware, most notably the widespread availability of GPUs, coupled with continuous improvements in deep learning algorithms, have propelled CNNs to the forefront of computer vision research [53,54].

**Figure 2:**
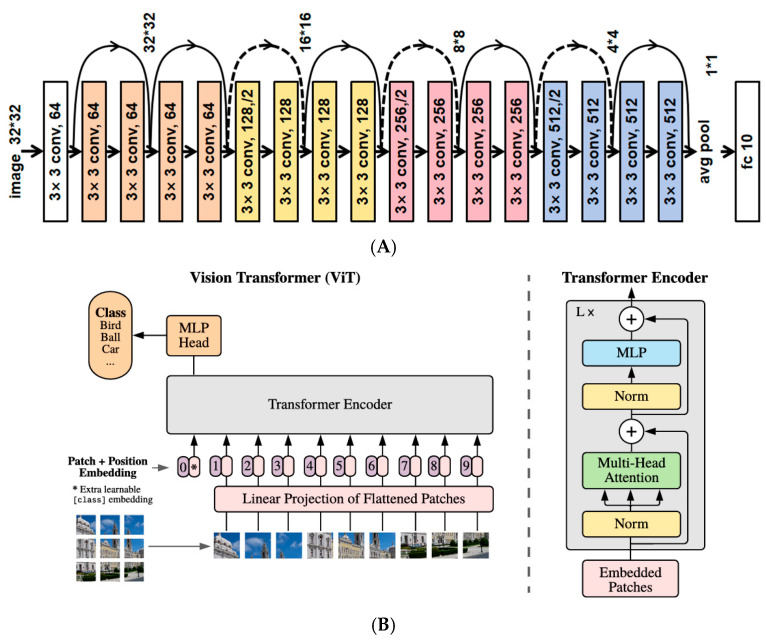
Comparative schematic of mainstream image classification frameworks: Residual Network (ResNet-18, (**A**)) vs. Vision Transformer (ViT, (**B**)).

In 2012, Krizhevsky et al. [55] introduced AlexNet, a groundbreaking CNN model that leveraged GPUs to significantly accelerate training processes. A critical innovation in their work was the emphasis on model depth, which proved essential for achieving superior performance in image classification tasks. The success of AlexNet, highlighted by its victory in the ImageNet competition, marked a transformative moment in artificial intelligence research [56]. An example of food image classification is presented in Figure 2, demonstrating the application of CNN-based models in distinguishing among various food types.

Building on these advancements, Riko Kusumoto and his team [57] extended the Bag-of-Features (BoF) model in 2014 by incorporating machine learning techniques based on sparse models and vector quantization. Their approach emphasized reconstructing local descriptors, significantly reducing the loss of image feature information and thereby improving feature extraction accuracy [58].

In 2017, Paritosh Pandey et al. [59] advanced CNN architectures by integrating AlexNet, GoogleNet, and ResNet into a novel multi-layer network. This innovative design exhibited exceptional performance on the ETH Food-101 dataset and a custom dataset focused on Indian cuisine [60], highlighting the effectiveness of combining diverse network architectures to enhance classification accuracy. Continuing this trajectory, in 2018, Martinel N and his team [61] developed a hybrid model that fused sliced convolutions with ResNet, specifically designed to capture vertical structural features in images of Western cuisine. Their model focused on accurately recognizing vertically structured dishes such as burgers, club sandwiches, multi-layered cakes, and lasagna—foods that pose particular classification challenges due to overlapping ingredients and inconsistent presentation. The proposed system was deployed in scenarios such as smart restaurant ordering systems, digital dietary tracking tools, and semi-automated kitchen monitoring, aiming to improve both accuracy and interpretability in real-world applications. Their approach achieved an impressive Top-1 accuracy of 90.27% on the Food-101 dataset, underscoring the potential of such innovations to significantly enhance image recognition performance [62]. Contemporary image classification methods primarily follow two competing paradigms: the CNN-based approach epitomized by ResNet and the Transformer-based approach pioneered by Vision Transformer (ViT). While demonstrating distinct characteristics in feature extraction, architecture design, and application domains, these approaches have recently converged through various hybrid architectures. They perform on the current largest food dataset, as shown in Table 3.

**b. Object Detection and Semantic Segmentation.**

In food-related scenarios, object detection and segmentation techniques enhance classification by distinguishing between overlapping and co-present food items. In agricultural domains, similar approaches have improved fruit detection, harvesting path planning, and crop row detection [77,78,79,80,81].

**Figure 3:**
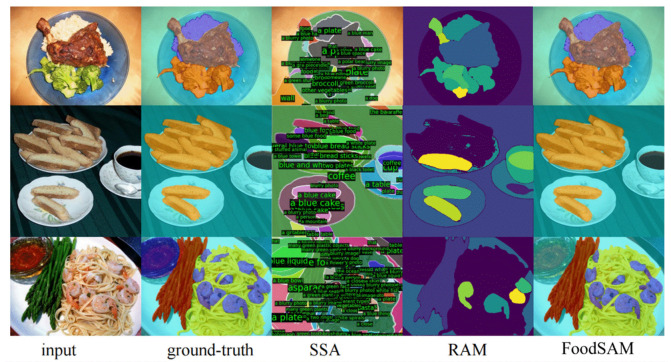
Food image segmentation benchmark: Ground-Truth vs. SSA vs. RAM vs. FoodSAM.

In nutrition monitoring, segmentation techniques enable precise identification of food components, supporting recipe generation and dietary guidance [82,83]. With the emergence of self-supervised models like SAM and BEIT, segmentation has become critical for enhancing food image interpretation (Figure 3).

**c. Knowledge Distillation and Few-Shot Learning.**

These approaches address computational and data scarcity challenges. Knowledge distillation transfers learning from large teacher models (e.g., ResNet-50) to smaller student models (e.g., VGG-16), preserving accuracy with lower complexity [84,85]. Few-shot learning helps classify new food types with minimal examples, ideal for dynamic or region-specific Chinese dishes.

**d. Monocular Depth Estimation.**

Volume estimation is critical for calorie assessment. Modern models use RGB-based monocular depth prediction via ViT, diffusion, and distillation models to infer food volumes, improving nutritional estimation over traditional 3D reconstruction techniques. Current state-of-the-art models achieve high precision in metric depth estimation (e.g., ZeroDepth [86] reduces scale ambiguity errors by 16.8% on the KITTI dataset, while Metric3D v2 [87] achieves 5% relative error without scale alignment). However, challenges persist in edge blurring and detail loss, especially for complex food geometries. For instance, PatchFusion improves resolution via tile-based fusion but requires 16–146× longer processing time than baseline methods, and diffusion-based models like Marigold suffer from temporal inconsistency in video applications [88,89].

#### 3.2.3. Special Challenges and Solutions in Chinese Food Image Classification

Despite significant progress in food image recognition, applying these methods to Chinese cuisine remains challenging due to limitations in existing datasets. Most public datasets are built from web-crawled images, which introduce two major issues:

Cross-domain and cross-category noise. Images retrieved online often include packaged foods, raw ingredients, or miscategorized dishes—introducing **semantic noise**. Many labeled categories contain visually inconsistent or irrelevant samples.

**Figure 4:**
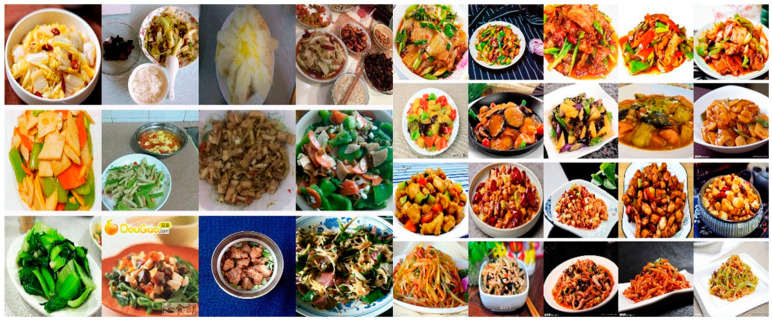
(**Left**): the noise in the currently available Chinese cuisine dataset, with the food names from top to bottom being stir-fried cabbage, oyster sauce king oyster mushrooms, and stir-fried vegetables. (**Right**): The same dish can appear differently due to variations in cooking methods or presentation angles. The food names from top to bottom are twice-cooked pork; stir-fried potato, pepper, and eggplant; Kung Pao chicken; and fish-flavored shredded pork.

Figure 4 **left** shows how noise affects typical categories like stir-fried cabbage or king oyster mushrooms. Moreover, dishes with the same name can look drastically different due to cooking variations or angles, leading to **cross-category confusion** (Figure 4 **right**).

**High redundancy and low quality** Many web images are near-duplicates, inflating dataset size without adding meaningful diversity. In addition, issues such as background clutter, lighting variation, and low resolution further hinder feature extraction.

**Lack of regional diversity** Chinese cuisine is deeply regional. Existing datasets often reflect narrow or localized samples, causing deep models to overfit superficial features rather than learn intrinsic visual patterns.

These limitations directly impact classification accuracy on Chinese-specific benchmarks, as shown in Table 4.

To overcome these challenges, future work should do the following: curate high-quality, regionally diverse datasets with expert labeling; apply noise filtering and de-duplication techniques during data preparation; use robust models that incorporate semantic segmentation or contextual learning; and explore few-shot learning for underrepresented or variant-rich dishes. These directions will improve recognition performance and make AI systems more adaptable to the rich diversity of Chinese cuisine.

## 4. Hyperspectral Imaging

Hyperspectral imaging (HSI) has emerged as a powerful non-destructive technique for food analysis, owing to its capacity to extract spectral–spatial fusion features that allow both qualitative and quantitative insights [96]. In this section, we explore the technical foundations, application scenarios, deep learning integration, and remaining challenges of HSI within the context of food—particularly Chinese cuisine. Rather than merely describing imaging technologies, this section aims to reveal how hyperspectral analysis resolves limitations of traditional food recognition while identifying key bottlenecks and future directions for intelligent food inspection [97].

### 4.1. Hyperspectral Imaging Techniques

Hyperspectral imaging has emerged as a powerful modality in food image analysis, offering advantages that go far beyond traditional imaging techniques. By capturing dense spectral information across the visible and near-infrared range, HSI enables precise identification of food attributes such as freshness, ripeness, moisture content, fat distribution, and contamination—attributes often invisible to the human eye or conventional RGB cameras [98]. This makes it particularly valuable for applications including non-destructive quality inspection, early spoilage detection, adulteration screening, and intelligent sorting in production lines. The integration of spectral and spatial features not only enhances classification accuracy but also enables pixel-level analysis [99], which is critical for assessing heterogeneous or visually similar food products. These capabilities have positioned HSI as a key enabler of intelligent, automated, and data-driven decision-making in modern agri-food systems.

#### 4.1.1. Hyperspectral Imaging Equipment and Technical Principles

Hyperspectral imaging captures three-dimensional data cubes (X-Y spatial axes and Z spectral axis), enabling highly detailed material analysis based on each substance’s spectral “fingerprint.” These fingerprints are formed through substance-specific reflection and absorption characteristics [100]—such as myoglobin oxidation peaks (660 nm) or chlorophyll absorption valleys (680 nm)—which serve as the physical foundation for non-invasive compositional detection [101].

To separate spectral components, systems use dispersive (high resolution), filtering (flexible), or interferometric (high SNR) methods. Most commercial systems adopt push-broom scanning, where line-array detectors acquire synchronized spectral slices during object displacement.

**Figure 5:**
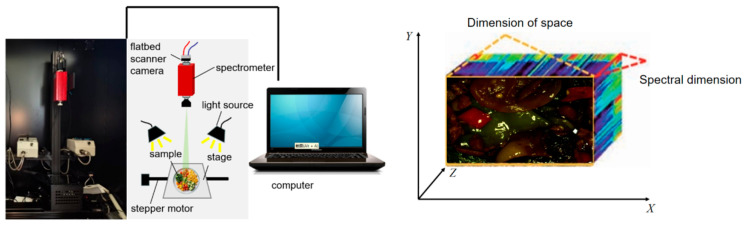
Hyperspectral camera schematic (**left**) and comparison of raw vs. smoothed spectral curves (**right**).

A typical HSI system comprises an optical module, detector, and data processing unit (Figure 5, left). In optical design, dispersive elements account for 30–50% of the system volume and require collaborative optimization with collimating and focusing lenses to enhance optical path efficiency [102]. The illumination module employs halogen lamps (covering 400–2500 nm broadband) or LED arrays (narrowband tunable) to ensure uniform lighting. Detector performance directly impacts detection sensitivity: silicon-based CCD/CMOS and InGaAs combinations are widely used in visible-shortwave infrared bands, achieving spatial resolutions up to 5 μm [103]. Push-broom scanning mode (adopted by 80% of commercial systems) dominates data acquisition, synchronizing line-array detectors with displacement stages to capture spatial–spectral information [104]. Snapshot techniques (e.g., coded aperture) improve frame rates but sacrifice resolution. Data processing involves three stages: radiometric correction (eliminating light source fluctuations), geometric correction (spatial registration error <0.1 pixel), and spectral unmixing (endmember extraction error <5%), supported by high-precision displacement stages (±0.1 mm accuracy) and large-capacity storage systems (single-scan data volume up to 200 GB) for large-scale experiments.

#### 4.1.2. Spectral Band Functionality and Food Inspection Applications

HSI enables **functional food analysis** across visible to shortwave infrared (400–2500 nm), where different bands correlate with specific physical or chemical attributes: Visible (400–760 nm): Blue (450–495 nm): detects foreign objects via reflectance differences. Green (495–570 nm): assesses chlorophyll peaks for vegetable freshness. Red (620–700 nm): identifies bruises or meat browning using myoglobin absorption.

Near to shortwave infrared (760–2500 nm): 780, 1450, and 1940 nm (OH bands): track moisture migration in baked goods. Values of 1724 and 1762 nm (CH_2_): detect lipid oxidation in meat. Values within the range of 1500–2300 nm (NH/CH): map proteins, starches, and carbohydrates.

These targeted spectral bands provide a solid engineering basis for building **portable, real-time food inspection devices**.

#### 4.1.3. Data Processing and Classification Enhancement

Unlike RGB-based methods, HSI excels in distinguishing visually similar objects through subtle spectral variances [105]. Enhanced data preprocessing—such as Savitzky–Golay smoothing or spectral unmixing—is essential for maintaining feature integrity. For instance, SG filtering with a 7–11 window achieves 92.3% feature retention under 40 dB SNR. Figure 5 (right) shows the improvement of spectral curve quality after smoothing, supporting more robust classification.

### 4.2. Deep Learning Approaches Based on Convolutional Neural Networks

#### 4.2.1. Background on Hyperspectral Analysis with CNNs

The success of AlexNet marked a pivotal shift in hyperspectral analysis, establishing convolutional neural networks (CNNs) as a foundational deep learning tool in this domain [106,107]. Early CNN-based models employed one-dimensional convolution layers, Batch Normalization, and PReLU activation to effectively extract spectral features, achieving promising classification accuracy even under limited training data conditions [108].

#### 4.2.2. Challenges in Modeling Complex Food Structures

While deep learning techniques have demonstrated strong performance in capturing spectral distinctions among food components, a critical limitation persists in modeling the underlying mechanisms that govern the discrimination of complex food structures. Specifically, many food products comprise multiple coexisting constituents—such as lipids, carbohydrates, and proteins—whose spectral features often exhibit significant overlap and nonlinear mixing [109]. In such cases, the spectral signatures do not correspond to isolated compounds but to complex, spatially intertwined matrices, making the interpretation of learned features inherently ambiguous. Current models predominantly rely on data-driven correlations without explicit consideration of biochemical interactions or physicochemical dependencies between constituents. As a result, although models may achieve high classification accuracy at the macro level, they often lack interpretability and robustness in tasks requiring fine-grained differentiation, such as estimating fat–protein ratios in emulsified products or distinguishing carbohydrate layers in cooked or processed foods.

Moreover, the low spatial resolution of hyperspectral data and the presence of spectral–spatial redundancy further complicate the accurate parsing of constituent-specific information. Existing fusion-based approaches—while improving robustness—do not fully resolve these ambiguities, particularly in scenarios involving heterogeneous, multi-phase food matrices. Therefore, the mechanistic basis by which deep networks differentiate among closely related or mixed spectral components remains an open problem, warranting further research in model interpretability, multi-modal integration, and constituent-level feature disentanglement.

#### 4.2.3. Representative Fusion Models

The low spatial resolution of hyperspectral data, combined with spectral–spatial redundancy, complicates the accurate extraction of constituent-specific information. Although existing fusion-based approaches have enhanced robustness, they fail to fully address these challenges, especially in scenarios involving heterogeneous, multi-phase food matrices [110]. As a result, the fundamental mechanisms by which deep learning networks distinguish between closely related or mixed spectral components remain unclear, highlighting the need for further research into model interpretability, multi-modal integration, and the disentangling of constituent-level features.

**Figure 6:**
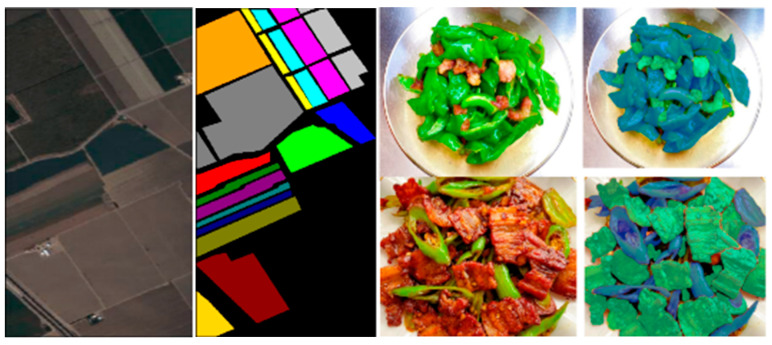
Hyperspectral image region segmentation of remote sensing (**left**) and dish (**right**).

To address these limitations, several spectral–spatial fusion models have been proposed, offering improvements in segmentation and classification tasks by associating spectral features (e.g., moisture, lipid content) with spatial patterns within food matrices [111]. However, their performance is still limited in highly complex or mixed food systems, signaling a clear need for biologically informed and interpretable modeling strategies. Figure 6 illustrates a comparison of HSI region segmentation between remote sensing tasks (left) and food applications (right), emphasizing the adaptability of these techniques across domains.

### 4.3. Challenges and Future Research Directions

Despite its potential, hyperspectral imaging faces several technical bottlenecks in food applications:

Data and computation efficiency: High-dimensional data increases memory and runtime requirements. Reducing algorithmic complexity while maintaining accuracy remains an urgent goal.

Training sample limitations: Many deep models rely on large, labeled datasets, which are difficult to obtain in food scenarios. Research into few-shot learning, data augmentation, and transfer learning is essential.

Model sparsity and deployment: lightweight models with high sparsity and robust performance are necessary for real-world use in handheld or embedded devices.

Classifier innovation: integrating multiple classifiers or novel architectures could enhance accuracy and stability in complex food environments.

Future work must balance precision, efficiency, and generalization to move hyperspectral food recognition from lab settings toward scalable, real-time deployment.

## 5. Application of Hyperspectral Technology in Food Inspection

Hyperspectral imaging (HSI) has become a transformative tool in modern food inspection due to its ability to conduct non-destructive, real-time, and composition-sensitive analysis. By capturing rich spectral–spatial information, HSI goes beyond traditional surface imaging and offers robust solutions for food classification, quality control, and safety monitoring. This section explores both foundational and emerging applications of HSI in food detection, emphasizing its integration with AI and the trajectory for future innovation.

### 5.1. Foundations of Hyperspectral Technology in Food Detection

#### 5.1.1. Principles and Analytical Methods

At its core, HSI measures the spectral reflectance of materials, generating rich datasets that support both qualitative identification (e.g., variety classification) and quantitative evaluation (e.g., moisture or sugar content). This spectral-based approach enables inspection without damaging the food, making it well-suited for scenarios such as type recognition, freshness grading, and quality assessment.

**Figure 7:**
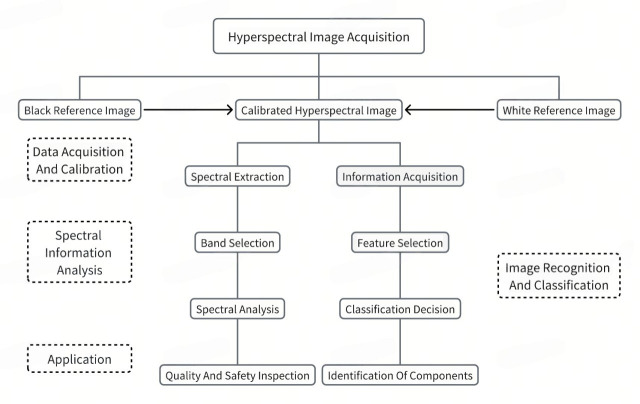
Application of hyperspectral technology in food detection.

Figure 7 illustrates major use cases where HSI has been deployed in non-destructive food detection systems—from surface defect detection to internal spoilage assessment.

#### 5.1.2. Empirical Applications

Hyperspectral imaging has been widely applied in diverse food detection scenarios, owing to its ability to extract rich spectral–spatial features non-destructively. For instance, it has been used to classify different varieties of vinegar and corn seeds [112] based on subtle spectral differences. Wang Jun proposed the Residual Attention Hierarchical Regression Network (RA-HRNet), which enhanced image reconstruction performance while reducing computational complexity, achieving 94.7% accuracy in identifying brewing sorghum varieties [113].

In food quality inspection, hyperspectral imaging has enabled accurate prediction of deoxynivalenol (DON) contamination levels in wheat flour [114], estimation of soluble solid content (SSC) in apples [115], and evaluation of sugar and moisture content in snow pears [116]. The technology has also proven effective in diagnosing nutrient deficiencies in plants, such as nitrogen, phosphorus, and potassium imbalance in tomato leaves [117].

Freshness discrimination is another key application area. Near-infrared hyperspectral imaging has demonstrated high accuracy in detecting internal mold in peanuts and differentiating freshness levels in pork, beef, and fish products based on biochemical markers such as myoglobin oxidation [118]. A detailed summary of these applications is presented in Table 5.

### 5.2. Emerging Applications Enabled by Hyperspectral and AI Technologies

Although hyperspectral imaging (HSI) has been widely applied in conventional food quality inspection, recent advances in artificial intelligence (AI), sensor fusion, and high-resolution imaging have significantly expanded its scope. These developments have enabled novel applications in intelligent food analysis, particularly in the prediction of nutritional value and spoilage levels in complex food matrices.

#### 5.2.1. Nutritional Monitoring: Semantic Segmentation and Deep Estimation

Deep learning algorithms, particularly those based on semantic segmentation, have facilitated fine-grained, pixel-level analysis of heterogeneous food compositions. Models such as the residual U-Net architecture have demonstrated efficacy in differentiating between food categories (e.g., meats, vegetables, and cereals) within a single dish. By isolating and characterizing each component, these models allow for accurate nutritional profiling.

Advanced systems—such as the CNTA’s “Food Safety 4.0” platform—combine HSI with deep learning to infer macronutrient distributions in real time. Empirical results suggest that such systems achieve up to a 40% reduction in estimation error compared to conventional image-based calorie estimation approaches. In addition, the integration of 3D food reconstruction and real-time nutritional databases enables more accurate caloric assessments, with demonstrated accuracy in estimating values (e.g., 215 kcal for mixed portions) even in diverse meal contexts.

Nonetheless, these technologies face persistent challenges in analyzing complex dishes, particularly those with overlapping or occluded ingredients. Such scenarios introduce spectral mixing and shape distortion, complicating the isolation of individual food items. Ongoing research addresses these limitations through the application of multi-spectral or hyper-multi-spectral fusion techniques, aiming to improve discrimination accuracy under non-ideal conditions.

#### 5.2.2. Intelligent Storage and Spoilage Detection

Recent innovations in smart food storage leverage hyperspectral and multi-sensor technologies for dynamic monitoring of food degradation processes. Smart refrigerators, such as the Meiling CHiQ series, employ image recognition systems and embedded databases to identify over 500 food types, track storage durations, and generate real-time spoilage alerts. Other systems, like those developed by Hisense, incorporate RFID tagging and load-cell-based weight sensors for automatic inventory tracking and lifecycle prediction.

**Figure 8:**
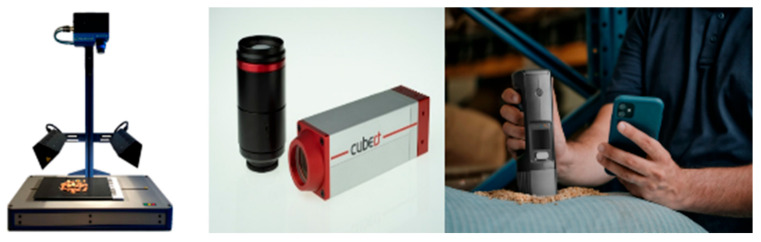
Representative portable hyperspectral detection devices.

From a biochemical standpoint, HSI enables non-destructive quantification of spoilage indicators—such as chlorophyll degradation in vegetables or protein breakdown in meats—by detecting subtle changes in reflectance spectra associated with moisture loss, microbial activity, and oxidation. Despite these advances, accurately assessing the freshness of unpackaged or non-standardized foods remains a technical hurdle. Current research focuses on machine learning-enhanced spectral interpretation, which seeks to improve model robustness across variable conditions, including lighting, packaging interference, and food heterogeneity. Figure 8 presents representative portable hyperspectral detection devices used in supporting these freshness and storage systems.

#### 5.2.3. Personalized Diets and Automated Serving Robots

**Existing cross-modal systems leverage** CNNs and large food databases to provide personalized dietary recommendations. **For example,** HealthBenefit’s system **identifies** dish components **and tailors** nutrition advice to individual health profiles.

**Current AI applications, such as** Cal AI’s system, **use** image recognition and LLMs to generate health-specific recipes. **Robotics in this domain has progressed from** simple tasks to full-process automation, **with developed systems like** Sweeper and LAVA **achieving** high precision in harvesting and cooking tasks.

### 5.3. Summary, Limitations, and Future Directions

Hyperspectral imaging has substantially enhanced food inspection capabilities, enabling high-precision detection of composition and freshness, intelligent food tracking, and personalized health interventions. Its integration with AI facilitates context-aware, data-driven decision-making across the entire food lifecycle.

However, several technical and practical challenges persist: **Data limitations**: existing datasets remain small, insufficiently diverse, or biased toward specific food types and imaging conditions. **Computational burden**: current models are resource-intensive, posing deployment challenges in edge and embedded environments. **Limited model generalization**: performance often deteriorates under real-world conditions, such as complex plating, poor lighting, and ingredient occlusion.

To address these challenges, future research should focus on the following: **Expanding hyperspectral datasets** to include richer annotations, a broader range of food categories, and more representative imaging conditions. **Integrating multi-modal sensing technologies**, such as NIR, Raman, thermal, and depth imaging, to enrich data diversity and improve recognition accuracy. **Developing lightweight models** capable of efficient inference on mobile and embedded systems. **Exploring omics-level data fusion**, combining spectral information with genomic, metabolomic, or microbiome data to enable truly personalized nutrition interventions.

Looking forward, advancements in food image recognition—particularly for complex and culturally rich cuisines like Chinese food—should prioritize the following key directions:

(1) **Construction of Multimodal Datasets:** To better capture the diversity and cultural context of Chinese cuisine, future datasets should integrate heterogeneous data types, including spectral and RGB images, nutritional information, textual labels (e.g., ingredient lists, dish names), and regional or cultural annotations. Developing automated annotation systems will be crucial to reducing labeling costs and facilitating the creation of large-scale, high-quality datasets that are both representative and generalizable across geographic regions.
(2) **Optimization of Deep Learning Models:** Achieving high classification accuracy under real-world constraints requires models that balance precision with efficiency. Research should emphasize the following: Lightweight neural networks and sparse classifiers suitable for mobile and embedded devices. Efficient spectral–spatial feature fusion techniques. Few-shot and data-efficient learning algorithms that perform well with limited samples.

Such models will enhance usability in resource-constrained environments, including handheld devices and low-power consumer electronics.

(3) **Advanced Applications of Hyperspectral Technology:** Beyond classification, **hyperspectral** imaging should be further leveraged for food safety and health monitoring, particularly in detecting trace elements, heavy metals, pesticide residues, and foodborne allergens or contaminants. These expanded applications would significantly enhance the practical impact of HSI in daily food inspection and public health assurance.
(4) **Development of Cross-Cultural and Generalizable Models:** To support global food AI applications, recognition systems must adapt across regions and cultures. This necessitates the following: Aggregating datasets representing diverse food traditions. Training models on culturally heterogeneous data. Incorporating knowledge transfer techniques to bridge gaps between different cuisines

Such efforts will facilitate the digital preservation and global dissemination of Chinese culinary heritage while also enabling cross-border applications in nutrition research, ingredient authentication, and dietary personalization.

In conclusion, this review underscores that food image recognition, particularly in the context of Chinese cuisine, requires solutions that transcend traditional visual modeling. As multimodal data integration, AI model optimization, and hyperspectral sensing technologies continue to mature, they will form a comprehensive framework for intelligent food analysis. With ongoing research into data diversity, algorithmic innovation, and cross-domain deployment, these technologies will not only enable smarter dietary recommendations and enhanced food safety management but also offer new methodologies for related domains such as ingredient quality grading, freshness monitoring, and cooking process recognition. Ultimately, these advancements will contribute to healthier lifestyles, safer food systems, and a deeper scientific understanding of food.

## 6. Conclusions

This review has systematically examined the evolution and current landscape of food image recognition technologies, with a particular emphasis on the classification and detection of Chinese cuisine images. The discussion traced progress from early RGB-based deep learning frameworks—such as convolutional neural networks (CNNs) and transfer learning models—to emerging hyperspectral imaging (HSI) approaches that aim to overcome inherent limitations in identifying visually complex, culturally diverse, and compositionally intricate food items.

While RGB-based methods have achieved moderate success in structured classification tasks, their performance remains constrained by several key factors, including high visual similarity between distinct dishes, sensitivity to environmental variables (e.g., lighting, occlusion), and limited generalization across dish variants. These limitations are especially pronounced in the context of Chinese cuisine, which is characterized by a vast array of regional styles, diverse ingredient combinations, and nuanced preparation techniques.

Hyperspectral imaging, with its capacity to capture rich spectral signatures at the material level, provides a non-destructive means to analyze food properties such as freshness, nutritional composition, and physicochemical quality. The fusion of HSI with advanced deep learning techniques—including knowledge distillation, few-shot learning, and spectral–spatial feature extraction—has significantly enhanced classification accuracy and expanded the applicability of food recognition systems beyond laboratory conditions.

Nevertheless, several challenges remain. These include the scarcity of large-scale, annotated hyperspectral food datasets, the computational demands of high-dimensional spectral modeling, and the discrepancy between controlled experimental settings and complex real-world deployment scenarios. In particular, Chinese cuisine presents unique challenges due to its ingredient-level heterogeneity, frequent use of mixed and overlapping components, and the prevalence of sauces and thermal transformations that obscure visual and spectral cues. The intrinsic complexity of such dishes—where a single plate may comprise multiple ingredients, each undergoing different preparation processes—renders conventional recognition and detection algorithms insufficient. Addressing these challenges will require more robust, context-aware models that can disentangle overlapping signals and adapt to the dynamic, culturally embedded nature of food presentation in Chinese culinary contexts.

## Figures and Tables

**Table 1:** Classification of common allergens in Chinese cuisine.

Allergen Category	Common Food Examples	Typical Chinese Cuisine Applications	Major Symptoms
Crustaceans/Fish	Shrimp, Crab, Perch	Seafood Congee, Steamed Fish, Spicy Hot Pot	Difficulty breathing, Laryngeal edema
Cereals	Wheat, Oats, Soy Sauce	Noodles, Steamed Buns, Stir-fry Seasoning	Digestive discomfort, Skin rash
Legumes/Nuts	Peanuts, Soybeans, Cashews	Kung Pao Chicken, Mapo Tofu, Cold Dishes	Anaphylactic shock, Abdominal pain
Dairy/Eggs	Milk, Eggs, Lactose	Desserts, Egg Dumplings, Milk Tea	Vomiting, Hives
Fruits	Mango, Pineapple, Strawberry	Desserts, Sweet and Sour Dishes	Stomatitis, Lip swelling

**Table 2:** Dish image datasets.

Dataset Name	Year	Images/Classes	Source	Coverage
UECFOOD-100 [34]	2012	14,361/100	Web	Japanese
Food-101 [35]	2014	10,100/101	Food spotting	Western
Vegfru [36]	2017	160,000/292	Web	Misc.
FoodX-251 [37]	2019	158,846/251	Web	Misc.
MyFoodRepo-273 [38]	2022	24,119/273	Web	Misc.
Food2k [39]	2023	1,036,564/2000	Web	Misc.
ISIA Food-500	2020	399,726/500	Web	Misc.
FoodSeg103 [40]	2024	7118/104	Recipe1M	Misc.
VIREO Food-172 [41]	2016	110,241/172	Web	Chinese
ChineseFoodNet [42]	2017	185,628/208	Web, Recipe, Menu	Chinese
CNFOOD-241 [43]	2023	191,811/241	Web	Chinese

**Table 3:** Top-1 and Top-5 accuracy of various image classification models on the Food2K dataset.

Model	Food2K	
	Top-1 Acc.	Top-5 Acc.
VGG-16 [63]	78.96	85.94
ResNet152 [64]	81.95	96.57
Inception-ResNet-v4 [65]	82.07	96.74
WRN-50–2-bottleneck [66]	81.94	96.19
DenseNet161 [67]	81.87	96.53
SE-ResNeXt101_32x4d [68]	80.81	95.61
SENet154 [68]	83.62	97.22
NTS-NET (ResNet50) [69]	81.24	94.94
HBP (ResNet50) [70]	77.56	92.87
WS-DAN (ResNet50) [71]	81.37	96.27
Inception v4 @ 448px [65]	82.46	97.17
MOMN (ResNet50) [72]	80.84	96.02
PMG (ResNet50) [73]	81.29	96.12
DLA [74]	80.14	96.37
PAR-Net (ResNet101) [75]	80.93	96.6
PRENet (ResNet101) [39]	83.75	97.33
Ensemble (Inception_v4, Swin_S, ViT_B, MViTv2_B) [76]	86.22	98.04

**Table 4:** Performance comparison of state-of-the-art models on Chinese food recognition benchmarks (CNFOOD-241).

Model	Year	Pretrain Weights	CNFOOD-241	
			Top-1 Acc.	Top-5 Acc.
VGG-16 [63]	2014	Y	64.05	85.94
GoogLeNet [90]	2014	Y	67.49	88.45
ResNet-101 [64]	2015	Y	69.58	92.44
DesNet-121 [67]	2016	Y	73.62	93.87
MobilNetV3 [91]	2017	Y	66.74	86.74
EfficientNetB6 [92]	2019	Y	78.07	95.41
ViT-B/16 [93]	2020	Y	69.75	92.52
Swin Transformer [94]	2021	Y	80.02	95.69
Res-Vmamba [95]	2024	Y	82.15	96.91
Ensemble (ResNeXt101@448, VOLO_D3, MViTv2_B_KD) [76]	2025	Y	83.09	97.29

**Table 5:** Application of hyperspectral technology in non-destructive testing of food.

Product	Application	Accuracy (Based on the Latest)	References
Fresh meat	Total bacterial count	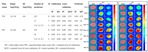	[119,120,121,122]
	Biogenic amine index	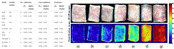	[123,124]
	Quality sorting	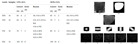	[125,126,127,128,129,130,131,132,133]
	Fecal contamination	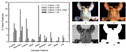	[134]
	Spoilage	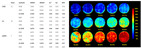	[135,136,137,138,139,140,141]
	Protein content	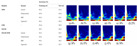	[142,143]
Fruits and vegetables [18]	Pesticide residues	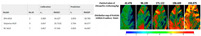	[144,145]
	Trace Elements and sugar content	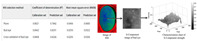	[146,147,148,149]
	Variety identification	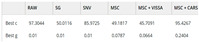	[150,151,152,153,154,155,156]
	Water content	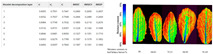	[157,158]
	Spoilage	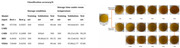	[159]
	Heavy metal	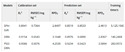	[160,161,162,163,164]
	Soluble solid content	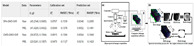	[165,166]
Meat products	Adulteration	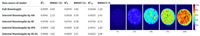	[167,168]
	Foreign body	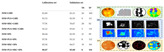	[169]
Tea and coffee	Breed identification	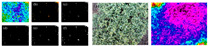	[170,171,172]
	Determination of mold	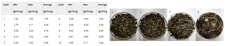	[173]
	Quality	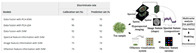	[174,175,176]
	Fumigated and dyed	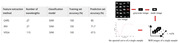	[177]
	Water content	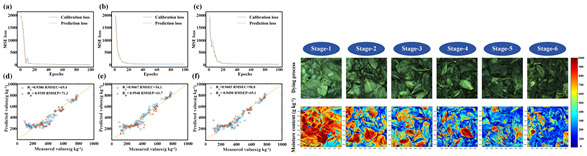	[178]

## Data Availability

No new data were created or analyzed in this study. Data sharing is not applicable to this article.

## References

[1] Chang Y. (2014). Research on Dietary Nutrition and Health Issues Among College Students. J. Heilongjiang Coll. Educ..

[2] Estay K., Proserpio C., Cattaneo C., Laureati M. (2025). Children’s food neophobia across different socioeconomic backgrounds in Chile: Exploring acceptance and willingness to try unfamiliar vegetables. Food Qual. Preference.

[3] Liu Y., Liu C., Sun L., Li M., Zhu Y., Deng W., Yu J., Zhang W., Song Z. (2025). Investigating flavor and quality characteristics in Chinese bacon from different regions using integrated GC-IMS, electronic sensory assessment, and sensory analysis. Meat Sci..

[4] Ding H., Tian J., Yu W., Wilson D.I., Young B.R., Cui X., Xin X., Wang Z., Li W. (2023). The application of artificial intelligence and big data in the food industry. Foods.

[5] Namkhah Z., Fatemi S.F., Mansoori A., Nosratabadi S., Ghayour-Mobarhan M., Sobhani S. (2023). Advancing sustainability in the food and nutrition system: A review of artificial intelligence applications. Front. Nutr..

[6] Raki H., Aalaila Y., Taktour A., Peluffo-Ordóñez D. (2023). Combining AI tools with non-destructive technologies for crop-based food safety: A comprehensive review. Foods.

[7] Min W., Jiang S., Liu L., Rui Y., Jain R. (2019). A survey on food computing. ACM Comput. Surv..

[8] Shen C., Wang R., Nawazish H., Wang B., Cai K., Xu B. (2024). Machine vision combined with deep learning–based approaches for food authentication: An integrative review and new insights. Compr. Rev. Food Sci. Food Saf..

[9] Kaushal S., Tammineni D.K., Rana P., Sharma M., Sridhar K., Chen H. (2024). Computer vision and deep learning-based approaches for detection of food nutrients/nutrition: New insights and advances. Trends Food Sci. Technol..

[10] Kim H., Venkataramanan R., Sheth A. (2024). A Survey on Food Ingredient Substitutions. arXiv.

[11] Wang C. (2019). Research on Food Intelligent Recognition Technology Based on Machine Vision.

[12] Li Y. (2022). Study on the Oil Content of Stir-Fried Dishes and the Degradation of Repeatedly Used Cooking Oil Quality.

[13] Minocha N., Singh A. (2025). Nutrition Essentials: Building a Foundation for Optimal Health Through Diet. Impact of Yoga and Proper Diet on Cardiopulmonary Function.

[14] Evert A.B., Dennison M., Gardner C.D., Garvey W.T., Lau K.H.K., MacLeod J., Mitri J., Pereira R.F., Rawlings K., Robinson S. (2019). Nutrition therapy for adults with diabetes or prediabetes: A consensus report. Diabetes Care.

[15] Mousavi Khaneghah A., Mostashari P. (2025). Decoding food reactions: A detailed exploration of food allergies vs. intolerances and sensitivities. Crit. Rev. Food Sci. Nutr..

[16] Ding H.H., Xie Z.Q., Yu W., Cui X.H., Wilson D.I. (2025). Artificial intelligence enhances food testing process: A comprehensive review. Food Biosci..

[17] Gu C.Y., Wang G., Zhuang W.H., Hu J., He X., Zhang L., Du Z., Xu X.M., Yin M.G., Yao Y.C. (2025). Artificial intelligence-enabled analysis methods and their applications in food chemistry. Crit. Rev. Food Sci. Nutr..

[18] Yang C., Guo Z., Fernandes Barbin D., Dai Z., Watson N., Povey M., Zou X. (2025). Hyperspectral Imaging and Deep Learning for Quality and Safety Inspection of Fruits and Vegetables: A Review. J. Agric. Food Chem..

[19] Siar M., Teshnehlab M. (2022). A combination of feature extraction methods and deep learning for brain tumour classification. IET Image Process..

[20] Sun D.-W., Pu H., Yu J. (2024). Applications of hyperspectral imaging technology in the food industry. Nat. Rev. Electr. Eng..

[21] Coman L.-I., Ianculescu M., Paraschiv E.-A., Alexandru A., Bădărău I.-A. (2024). Smart Solutions for Diet-Related Disease Management: Connected Care, Remote Health Monitoring Systems, and Integrated Insights for Advanced Evaluation. Appl. Sci..

[22] Lu B., Dao P.D., Liu J., He Y., Shang J. (2020). Recent advances of hyperspectral imaging technology and applications in agriculture. Remote. Sens..

[23] Cao R., Li J., Ding H., Zhao T., Guo Z., Li Y., Sun X., Wang F., Qiu J. (2024). Synergistic approaches of AI and NMR in enhancing food component analysis: A comprehensive review. Trends Food Sci. Technol..

[24] Huang W., Yin M., Xia J., Zhang X. (2024). A review of cross-scale and cross-modal intelligent sensing and detection technology for food quality: Mechanism analysis, decoupling strategy and integrated applications. Trends Food Sci. Technol..

[25] Guo B., Lu X., Jiang X., Shen X.-L., Wei Z., Zhang Y.J.F. (2025). Artificial Intelligence in Advancing Algal Bioactive Ingredients: Production, Characterization, and Application. Foods.

[26] Ma P.H., Lau C.P., Yu N., Li A., Liu P., Wang Q., Sheng J.P. (2021). Image-based nutrient estimation for Chinese dishes using deep learning. Food Res. Int..

[27] Zhou J., Xin X., Li W., Ding H.H., Yu S., Cui X.H. (2024). Flavor analysis and region prediction of Chinese dishes based on food pairing. Inf. Process. Manag..

[28] Schmidgall S., Ziaei R., Achterberg J., Kirsch L., Hajiseyedrazi S., Eshraghian J. (2024). Brain-inspired learning in artificial neural networks: A review. APL Mach. Learn..

[29] Hammad M. (2024). Deep Learning Activation Functions: Fixed-Shape, Parametric, Adaptive, Stochastic, Miscellaneous, Non-Standard, Ensemble. arXiv.

[30] Nwankpa C., Ijomah W., Gachagan A., Marshall S. (2018). Activation functions: Comparison of trends in practice and research for deep learning. arXiv.

[31] Dubey S.R., Singh S.K., Chaudhuri B.B. (2022). Activation functions in deep learning: A comprehensive survey and benchmark. Neurocomputing.

[32] Aliferis C., Simon G. (2024). Overfitting, underfitting and general model overconfidence and under-performance pitfalls and best practices in machine learning and AI. Artif. Intell. Mach. Learn. Health Care Med. Sci. Best Pract. Pitfalls.

[33] Bidyalakshmi T., Jyoti B., Mansuri S.M., Srivastava A., Mohapatra D., Kalnar Y.B., Narsaiah K., Indore N. (2024). Application of Artificial Intelligence in Food Processing: Current Status and Future Prospects. Food Eng. Rev..

[34] Matsuda Y., Yanai K. Multiple-food recognition considering co-occurrence employing manifold ranking. Proceedings of the 21st International Conference on Pattern Recognition (ICPR2012).

[35] Bossard L., Guillaumin M., Gool L.V. Food-101-Mining Discriminative Components with Random Forests. Proceedings of the 13th European Conference on Computer Vision (ECCV).

[36] Hou S.H., Feng Y.S., Wang Z.L. VegFru: A Domain-Specific Dataset for Fine-grained Visual Categorization. Proceedings of the 16th IEEE International Conference on Computer Vision (ICCV).

[37] Kaur P., Sikka K., Wang W., Belongie S., Divakaran A. (2019). Foodx-251: A dataset for fine-grained food classification. arXiv.

[38] Mohanty S.P., Singhal G., Scuccimarra E.A., Kebaili D., Heritier H., Boulanger V., Salathe M. (2022). The Food Recognition Benchmark: Using Deep Learning to Recognize Food in Images. Front. Nutr..

[39] Min W.Q., Wang Z.L., Liu Y.X., Luo M.J., Kang L.P., Wei X.M., Wei X.L., Jiang S.Q. (2023). Large Scale Visual Food Recognition. IEEE Trans. Pattern Anal. Mach. Intell..

[40] Wu X., Fu X., Liu Y., Lim E.-P., Hoi S.C.H., Sun Q. A large-scale benchmark for food image segmentation. Proceedings of the 29th ACM International Conference on Multimedia.

[41] Chen J.J., Ngo C.W. Deep-based Ingredient Recognition for Cooking Recipe Retrieval. Proceedings of the 24th ACM Multimedia Conference (MM).

[42] Chen X., Zhu Y., Zhou H., Diao L., Wang D. (2017). Chinesefoodnet: A large-scale image dataset for chinese food recognition. arXiv.

[43] Fan B.K., Li W.Q., Dong L., Li J.Z., Nie Z.D. Automatic Chinese Food recognition based on a stacking fusion model. Proceedings of the 45th Annual International Conference of the IEEE-Engineering-in-Medicine-and-Biology-Society (EMBC).

[44] Zayas I. (1985). Discrimination between Arthur and Arkan wheats by image analysis. Cereal Chem..

[45] Lai F.S., Zayas I., Pomeranz Y. (1986). Application of pattern recognition techniques in the analysis of cereal grains. Cereal Chem..

[46] Deng Z., Wang T., Zheng Y., Zhang W., Yun Y.-H. (2024). Deep learning in food authenticity: Recent advances and future trends. rends Food Sci. Technol..

[47] Xia B., Abidin M.R.Z., Ab Karim S. (2024). From tradition to technology: A comprehensive review of contemporary food design. Int. J. Gastron. Food Sci..

[48] Khan A., Sohail A., Zahoora U., Qureshi A.S. (2020). A survey of the recent architectures of deep convolutional neural networks. Artif. Intell. Rev..

[49] Sarraf A., Azhdari M., Sarraf S. (2021). A comprehensive review of deep learning architectures for computer vision applications. Am. Sci. Res. J. Eng. Technol. Sci. ASRJETS.

[50] Jiang C., Zhou Q., Lei J., Wang X. (2022). A Two-Stage Structural Damage Detection Method Based on 1D-CNN and SVM. Appl. Sci..

[51] Özcan R., Tütüncü K., Karaca M. (2022). Comparison of Plant Detection Performance of CNN-based Single-stage and Two-stage Models for Precision Agriculture. Appl. Sci..

[52] Mao Y., Yu X., Huang K., Zhang Y.-J.A., Zhang J. (2024). Green edge AI: A contemporary survey. arXiv.

[53] Iqbal U., Davies T., Perez P. (2024). A Review of Recent Hardware and Software Advances in GPU-Accelerated Edge-Computing Single-Board Computers (SBCs) for Computer Vision. Sensors.

[54] Wang C. (2023). GPU-Based Acceleration and Optimization Research on Computer Vision. Ph.D. Dissertation.

[55] Krizhevsky A., Sutskever I., Hinton G.E. (2017). ImageNet Classification with Deep Convolutional Neural Networks. Commun. ACM.

[56] Rai D.H. (2024). Artificial Intelligence Through Time: A Comprehensive Historical Review. Bachelor’s Thesis.

[57] Kusumoto R., Han X.H., Chen Y.W.J.I. Sparse model in hierarchic spatial structure for food image recognition. Proceedings of the 2013 6th International Conference on Biomedical Engineering and Informatics.

[58] Romero A., Gatta C., Camps-Valls G. (2015). Unsupervised deep feature extraction for remote sensing image classification. IEEE Trans. Geosci. Remote. Sens..

[59] Pandey P., Deepthi A., Mandal B., Puhan N.B. (2017). FoodNet: Recognizing Foods Using Ensemble of Deep Networks. IEEE Signal Process. Lett..

[60] Sultana J., Ahmed B.M., Masud M.M., Huq A.O., Ali M.E., Naznin M. (2023). A study on food value estimation from images: Taxonomies, datasets, and techniques. IEEE Access.

[61] Martinel N., Foresti G.L., Micheloni C. Wide-Slice Residual Networks for Food Recognition. Proceedings of the 2018 IEEE Winter Conference on Applications of Computer Vision (WACV).

[62] Kiourt C., Pavlidis G., Markantonatou S. (2020). Deep learning approaches in food recognition. Machine Learning Paradigms: Advances in Deep Learning-Based Technological Applications.

[63] Simonyan K., Zisserman A. (2014). Very deep convolutional networks for large-scale image recognition. arXiv.

[64] He K.M., Zhang X.Y., Ren S.Q., Sun J. Deep Residual Learning for Image Recognition. Proceedings of the 2016 IEEE Conference on Computer Vision and Pattern Recognition (CVPR).

[65] Szegedy C., Ioffe S., Vanhoucke V., Alemi A.A. Inception-v4, Inception-ResNet and the Impact of Residual Connections on Learning. Proceedings of the 31st AAAI Conference on Artificial Intelligence.

[66] Alaeddine H., Jihene M. (2023). Wide deep residual networks in networks. Multimed. Tools Appl..

[67] Huang G., Liu Z., van der Maaten L., Weinberger K.Q. Densely Connected Convolutional Networks. Proceedings of the 30th IEEE/CVF Conference on Computer Vision and Pattern Recognition (CVPR).

[68] Hu J., Shen L., Albanie S., Sun G., Wu E.H. (2020). Squeeze-and-Excitation Networks. IEEE Trans. Pattern Anal. Mach. Intell..

[69] Yang Z., Luo T.G., Wang D., Hu Z.Q., Gao J., Wang L.W. Learning to Navigate for Fine-Grained Classification. Proceedings of the 15th European Conference on Computer Vision (ECCV).

[70] Yu C.J., Zhao X.Y., Zheng Q., Zhang P., You X.G. Hierarchical Bilinear Pooling for Fine-Grained Visual Recognition. Proceedings of the 15th European Conference on Computer Vision (ECCV).

[71] Hu T., Qi H., Huang Q., Lu Y. (2019). See better before looking closer: Weakly supervised data augmentation network for fine-grained visual classification. arXiv.

[72] Min S.B., Yao H.T., Xie H.T., Zha Z.J., Zhang Y.D. (2020). Multi-Objective Matrix Normalization for Fine-Grained Visual Recognition. IEEE Trans. Image Process..

[73] Du R., Chang D., Bhunia A.K., Xie J., Ma Z., Song Y.-Z., Guo J. Fine-grained visual classification via progressive multi-granularity training of jigsaw patches. Proceedings of the European Conference on Computer Vision.

[74] Yu F., Wang D.Q., Shelhamer E., Darrell T. Deep Layer Aggregation. Proceedings of the 31st IEEE/CVF Conference on Computer Vision and Pattern Recognition (CVPR).

[75] Qiu J., Lo F.P.-W., Sun Y., Wang S., Lo B. (2022). Mining discriminative food regions for accurate food recognition. arXiv.

[76] Nong L., Peng G., Xu T., Zhu J.J.E.A.o.A.I. (2025). From ensemble to knowledge distillation: Improving large-scale food recognition. Eng. Appl. Artif. Intell..

[77] Ji W., Zhang T., Xu B., He G.Z. (2024). Apple recognition and picking sequence planning for harvesting robot in a complex environment. J. Agric. Eng..

[78] Garcia-Garcia A., Orts-Escolano S., Oprea S., Villena-Martinez V., Martinez-Gonzalez P., Garcia-Rodriguez J. (2018). A survey on deep learning techniques for image and video semantic segmentation. Appl. Soft Comput..

[79] Garcia-Garcia A., Orts-Escolano S., Oprea S., Villena-Martinez V., Garcia-Rodriguez J. (2017). A review on deep learning techniques applied to semantic segmentation. arXiv.

[80] Shang G., Liu G., Zhu P., Han J., Xia C., Jiang K. (2020). A deep residual U-Type network for semantic segmentation of orchard environments. Appl. Sci..

[81] Anagnostis A., Tagarakis A.C., Kateris D., Moysiadis V., Sørensen C.G., Pearson S., Bochtis D.J.S. (2021). Orchard mapping with deep learning semantic segmentation. Sensors.

[82] Pfisterer K.J., Amelard R., Chung A.G., Syrnyk B., MacLean A., Keller H.H., Wong A. (2022). Automated food intake tracking requires depth-refined semantic segmentation to rectify visual-volume discordance in long-term care homes. Sci. Rep..

[83] Pfisterer K.J., Amelard R., Chung A.G., Syrnyk B., MacLean A., Keller H.H., Wong A.J. (2019). When segmentation is not enough: Rectifying visual-volume discordance through multisensor depth-refined semantic segmentation for food intake tracking in long-term care. arXiv.

[84] Shen Z., Savvides M.J. (2020). Meal v2: Boosting vanilla resnet-50 to 80%+ top-1 accuracy on imagenet without tricks. arXiv.

[85] Ye M., Ruiwen N., Chang Z., He G., Tianli H., Shijun L., Yu S., Tong Z., Ying G. (2021). A lightweight model of VGG-16 for remote sensing image classification. IEEE J. Sel. Top. Appl. Earth Obs. Remote. Sens..

[86] Guizilini V., Vasiljevic I., Chen D., Ambrus R., Gaidon A. Towards Zero-Shot Scale-Aware Monocular Depth Estimation. Proceedings of the IEEE/CVF International Conference on Computer Vision (ICCV).

[87] Hu M., Yin W., Zhang C., Cai Z.P., Long X.X., Chen H., Wang K.X., Yu G., Shen C.H., Shen S. (2024). Metric3D v2: A Versatile Monocular Geometric Foundation Model for Zero-Shot Metric Depth and Surface Normal Estimation. IEEE Trans. Pattern Anal. Mach. Intell..

[88] Ke B.X., Obukhov A., Huang S.Y., Metzger N., Daudt R.C., Schindler K. Repurposing Diffusion-Based Image Generators for Monocular Depth Estimation. Proceedings of the IEEE/CVF Conference on Computer Vision and Pattern Recognition (CVPR).

[89] Li Z., Bhat S.F., Wonka P. PatchFusion: An End-to-End Tile-Based Framework for High-Resolution Monocular Metric Depth Estimation. Proceedings of the IEEE/CVF Conference on Computer Vision and Pattern Recognition (CVPR).

[90] Szegedy C., Liu W., Jia Y.Q., Sermanet P., Reed S., Anguelov D., Erhan D., Vanhoucke V., Rabinovich A. Going Deeper with Convolutions. Proceedings of the IEEE Conference on Computer Vision and Pattern Recognition (CVPR).

[91] Howard A., Sandler M., Chu G., Chen L.C., Chen B., Tan M.X., Wang W.J., Zhu Y.K., Pang R.M., Vasudevan V. Searching for MobileNetV3. Proceedings of the IEEE/CVF International Conference on Computer Vision (ICCV).

[92] Tan M.X., Le Q.V. EfficientNet: Rethinking Model Scaling for Convolutional Neural Networks. Proceedings of the 36th International Conference on Machine Learning (ICML).

[93] Dosovitskiy A., Beyer L., Kolesnikov A., Weissenborn D., Zhai X., Unterthiner T., Dehghani M., Minderer M., Heigold G., Gelly S. (2020). An image is worth 16x16 words: Transformers for image recognition at scale. arXiv.

[94] Liu Z., Lin Y.T., Cao Y., Hu H., Wei Y.X., Zhang Z., Lin S., Guo B.N. Swin Transformer: Hierarchical Vision Transformer using Shifted Windows. Proceedings of the 18th IEEE/CVF International Conference on Computer Vision (ICCV).

[95] Chen C.-S., Chen G.-Y., Zhou D., Jiang D., Chen D.-S. (2024). Res-vmamba: Fine-grained food category visual classification using selective state space models with deep residual learning. arXiv.

[96] Wan G.L., He J.G., Meng X.H., Liu G.S., Zhang J.J., Ma F., Zhang Q., Wu D. (2025). Hyperspectral imaging technology for nondestructive identification of quality deterioration in fruits and vegetables: A review. Crit. Rev. Food Sci. Nutr..

[97] Nikzadfar M., Rashvand M., Zhang H.W., Shenfield A., Genovese F., Altieri G., Matera A., Tornese I., Laveglia S., Paterna G. (2024). Hyperspectral Imaging Aiding Artificial Intelligence: A Reliable Approach for Food Qualification and Safety. Appl. Sci..

[98] ElMasry G.M., Nakauchi S. (2016). Image analysis operations applied to hyperspectral images for non-invasive sensing of food quality—A comprehensive review. Biosyst. Eng..

[99] Liu Q.C., Xiao L., Yang J.X., Wei Z.H. (2021). CNN-Enhanced Graph Convolutional Network With Pixel- and Superpixel-Level Feature Fusion for Hyperspectral Image Classification. IEEE Trans. Geosci. Remote Sens..

[100] Soni A., Dixit Y., Reis M.M., Brightwell G. (2022). Hyperspectral imaging and machine learning in food microbiology: Developments and challenges in detection of bacterial, fungal, and viral contaminants. Compr. Rev. Food. Sci. Food Saf..

[101] Pu H.B., Lin L., Sun D.W. (2019). Principles of Hyperspectral Microscope Imaging Techniques and Their Applications in Food Quality and Safety Detection: A Review. Compr. Rev. Food. Sci. Food Saf..

[102] Kowalewski J., Domaradzki J., Zieba M., Podgórski M. (2023). Hyperspectral Imaging—A Short Review of Methods and Applications. Metrol. Meas. Syst..

[103] Wu Y., Wang Y.M., Zhang D. (2025). Design and Analysis of Spaceborne Hyperspectral Imaging System for Coastal Studies. Remote Sens..

[104] Arablouei R., Goan E., Gensemer S., Kusy B. Fast and robust pushbroom hyperspectral imaging via DMD-based scanning. Proceedings of the 19th Annual Conference on Novel Optical Systems Design and Optimization.

[105] Chang C.I. (2000). An information-theoretic approach to spectral variability, similarity, and discrimination for hyperspectral image analysis. IEEE Trans. Inf. Theory.

[106] Chen W., He J., Liu G. (2022). Hyperspectral Image Classification Based on Convolution Neural Network with Attention Mechanism. Laser Optoelectron. Prog..

[107] Wang A.L., Liu M.H., Xue D., Wu H.B., Zhao L.F., Yuji I. (2022). Hyperspectral Image Classification Combined Dynamic Convolution with Triplet Attention Mechanism. Laser Optoelectron. Prog..

[108] Mei S., Ji J., Bi Q., Hou J., Li W. Integrating spectral and spatial information into deep convolutional Neural Networks for hyperspectral classification. Proceedings of the 2016 IEEE International Geoscience and Remote Sensing Symposium (IGARSS).

[109] Karoui R., Downey G., Blecker C. (2010). Mid-Infrared Spectroscopy Coupled with Chemometrics: A Tool for the Analysis of Intact Food Systems and the Exploration of Their Molecular Structure-Quality Relationships—A Review. Chem. Rev..

[110] Guo M.Q., Wang K.Q., Lin H., Wang L., Cao L.M., Sui J.X. (2024). Spectral data fusion in nondestructive detection of food products: Strategies, recent applications, and future perspectives. Compr. Rev. Food. Sci. Food Saf..

[111] Tang S.Q., Zhong N., Zhou Y.H., Chen S.B., Dong Z.B., Qi L., Feng X. (2025). Synergistic spectral-spatial fusion in hyperspectral Imaging: Dual attention-based rice seed varieties identification. Food Control.

[112] Zhang L., Zhang S.B., Liu J.C., Wei Y.G., An D., Wu J.W. (2024). Maize seed variety identification using hyperspectral imaging and self-supervised learning: A two-stage training approach without spectral preprocessing. Expert Syst. Appl..

[113] Kohei Y., Han X.H., Ieee Comp S.O.C. Deep Residual Attention Network for Hyperspectral Image Reconstruction. Proceedings of the 25th International Conference on Pattern Recognition (ICPR).

[114] Almoujahed M.B., Apolo-Apolo O.E., Alhussein M., Kazlauskas M., Kriauciuniene Z., Sarauskis E., Mouazen A.M. (2025). Prediction of Deoxynivalenol contamination in wheat kernels and flour based on visible near-infrared spectroscopy, feature selection and machine learning modelling. Spectroc. Acta Pt. A-Molec. Biomolec. Spectr..

[115] Zhang D.Y., Xu Y.F., Huang W.Q., Tian X., Xia Y., Xu L., Fan S.X. (2019). Nondestructive measurement of soluble solids content in apple using near infrared hyperspectral imaging coupled with wavelength selection algorithm. Infrared Phys. Technol..

[116] Zhang Z.S., Cheng H., Chen M.Y., Zhang L.X., Cheng Y.D., Geng W.J., Guan J.F. (2024). Detection of Pear Quality Using Hyperspectral Imaging Technology and Machine Learning Analysis. Foods.

[117] Barbedo J.G.A. (2019). Detection of nutrition deficiencies in plants using proximal images and machine learning: A review. Comput. Electron. Agric..

[118] Shen X.Y., Xing L.J., Pan L.Q., Miao Y.J., Zhang W.G. (2025). Prediction of chicken breast meat freshness based on hyperspectral imaging technique and high-throughput sequencing. Poult. Sci..

[119] Barbin D.F., Elmasry G., Sun D.W., Allen P., Morsy N. (2013). Non-destructive assessment of microbial contamination in porcine meat using NIR hyperspectral imaging. Innov. Food Sci. Emerg. Technol..

[120] Feng Y.Z., Sun D.W. (2013). Determination of total viable count (TVC) in chicken breast fillets by near-infrared hyperspectral imaging and spectroscopic transforms. Talanta.

[121] Huang L., Zhao J., Zhang Y. (2020). Rapid detection of total viable count (TVC) in pork meat by hyperspectral imaging. Food Res. Int..

[122] Li H.H., Kutsanedzie F., Zhao J.W., Chen Q.S. (2016). Quantifying Total Viable Count in Pork Meat Using Combined Hyperspectral Imaging and Artificial Olfaction Techniques. Food Anal. Meth..

[123] Cheng W., Sun D.W., Cheng J.H. (2016). Pork biogenic amine index (BAI) determination based on chemometric analysis of hyperspectral imaging data. LWT.

[124] Yang D., Lu A., Ren D., Wang J. (2017). Rapid determination of biogenic amines in cooked beef using hyperspectral imaging with sparse representation algorithm. Infrared Phys. Technol..

[125] Liu L., Ngadi M.O., Prasher S.O., Gariepy C.J. (2010). Categorization of pork quality using Gabor filter-based hyperspectral imaging technology. J. Food Eng..

[126] Sun Z., Wang T., Zou X., Liu Y., Liang L., Li J., Liu X. (2021). Discrimination between Raw and Restructured Beef Steak Using Hyperspectral and Ultrasound Imaging. Food Sci..

[127] Sun Z.B., Pan H.D., Zuo M., Li J.K., Liang L.M., Ho C.T., Zou X.B. (2023). Non-destructive assessment of equivalent umami concentrations in salmon using hyperspectral imaging technology combined with multivariate algorithms. Spectroc. Acta Pt. A-Molec. Biomolec. Spectr..

[128] Yao K.S., Sun J., Chen C., Xu M., Cheng J.H., Zhou X. (2023). Non-Destructive Identification for Panax Notoginseng Powder of Different Parts Based on Hyperspectral Imaging Technique. Spectrosc. Spectr. Anal..

[129] Yao K.S., Sun J., Zhang L., Zhou X., Tian Y., Tang N.Q., Wu X.H. (2021). Nondestructive detection for egg freshness based on hyperspectral imaging technology combined with harris hawks optimization support vector regression. J. Food Saf..

[130] Yao K.S., Sun J., Zhou X., Nirere A., Tian Y., Wu X.H. (2020). Nondestructive detection for egg freshness grade based on hyperspectral imaging technology. J. Food Process Eng..

[131] Khulal U., Zhao J.W., Hu W.W., Chen Q.S. (2016). Nondestructive quantifying total volatile basic nitrogen (TVB-N) content in chicken using hyperspectral imaging (HSI) technique combined with different data dimension reduction algorithms. Food Chem..

[132] Xia Y., Xiao X.P., Adade S., Xi Q.B., Wu J., Xu Y., Chen Q.M., Chen Q.S. (2025). Physicochemical properties and gel quality monitoring of surimi during thermal processing using hyperspectral imaging combined with deep learning. Food Control.

[133] Li H.H., Li C.H., Shoaib M., Zhang W., Murugesan A. (2025). Advances in Non-Thermal Processing of Meat and Monitoring Meat Protein Gels Through Vibrational Spectroscopy. Foods.

[134] Park B., Lawrence K.C., Windham W.R., Buhr R.J. (2002). Hyperspectral imaging for detecting fecal and ingesta contamination on poultry carcasses. Trans. ASAE.

[135] Zhao H.-T., Feng Y.-Z., Chen W., Jia G.-F. (2019). Application of invasive weed optimization and least square support vector machine for prediction of beef adulteration with spoiled beef based on visible near-infrared (Vis-NIR) hyperspectral imaging. Meat Sci..

[136] Aheto J.H., Huang X.Y., Tian X.Y., Ren Y., Bonah E., Alenyorege E.A., Lv R.Q., Dai C.X. (2019). Combination of spectra and image information of hyperspectral imaging data for fast prediction of lipid oxidation attributes in pork meat. J. Food Process Eng..

[137] Cheng J.H., Sun J., Yao K.S., Dai C.X. (2023). Generalized and hetero two-dimensional correlation analysis of hyperspectral imaging combined with three-dimensional convolutional neural network for evaluating lipid oxidation in pork. Food Control.

[138] Sun Z., Liang L., Yan X., Zou X., Wang T., Liu X., Li J. (2020). Detection of Freshness Indexes of Imported Chilled Beef Using Hyperspectral Imaging Technology. Food Sci..

[139] Yao K.S., Sun J., Chen C., Xu M., Zhou X., Cao Y., Tian Y. (2022). Non-destructive detection of egg qualities based on hyperspectral imaging. J. Food Eng..

[140] Sun J., Cheng J.H., Xu M., Yao K.S. (2024). A method for freshness detection of pork using two-dimensional correlation spectroscopy images combined with dual-branch deep learning. J. Food Compos. Anal..

[141] Xi Q.B., Chen Q.M., Ahmad W., Pan J., Zhao S.G., Xia Y., Ouyang Q., Chen Q.S. (2025). Quantitative analysis and visualization of chemical compositions during shrimp flesh deterioration using hyperspectral imaging: A comparative study of machine learning and deep learning models. Food Chem..

[142] Sun J., Yang F.Y., Cheng J.H., Wang S.M., Fu L.H. (2024). Nondestructive identification of soybean protein in minced chicken meat based on hyperspectral imaging and VGG16-SVM. J. Food Compos. Anal..

[143] Yang F.Y., Sun J., Cheng J.H., Fu L.H., Wang S.M., Xu M. (2023). Detection of starch in minced chicken meat based on hyperspectral imaging technique and transfer learning. J. Food Process Eng..

[144] Jiang S.Y., Sun J., Xin Z., Mao H.P., Wu X.H., Li Q.L. (2017). Visualizing distribution of pesticide residues in mulberry leaves using NIR hyperspectral imaging. J. Food Process Eng..

[145] Sun J., Cong S.L., Mao H.P., Wu X.H., Yang N. (2018). Quantitative detection of mixed pesticide residue of lettuce leaves based on hyperspectral technique. J. Food Process Eng..

[146] Lu B., Sun J., Yang N., Hang Y.Y. (2019). Fluorescence hyperspectral image technique coupled with HSI method to predict solanine content of potatoes. J. Food Process Preserv..

[147] Lu X.Z., Sun J., Mao H.P., Wu X.H., Gao H.Y. (2017). Quantitative determination of rice starch based on hyperspectral imaging technology. Int. J. Food Prop..

[148] Shi L., Sun J., Cong S.L., Ji X.Y., Yao K.S., Zhang B., Zhou X. (2025). Fluorescence hyperspectral imaging for detection of selenium content in lettuce leaves under cadmium-free and cadmium environments. Food Chem..

[149] Yu K., Zhong M.M., Zhu W.J., Rashid A., Han R.W., Virk M.S., Duan K.W., Zhao Y.J., Ren X.F. (2025). Advances in Computer Vision and Spectroscopy Techniques for Non-Destructive Quality Assessment of *Citrus* Fruits: A Comprehensive Review. Foods.

[150] Ahmad H., Sun J., Nirere A., Shaheen N., Zhou X., Yao K.S. (2021). Classification of tea varieties based on fluorescence hyperspectral image technology and ABC-SVM algorithm. J. Food Process Preserv..

[151] Dai C.X., Sun J., Huang X.Y., Zhang X.R., Tian X.Y., Wang W., Sun J.T., Luan Y. (2023). Application of Hyperspectral Imaging as a Nondestructive Technology for Identifying Tomato Maturity and Quantitatively Predicting Lycopene Content. Foods.

[152] Sun J., Jiang S.Y., Mao H.P., Wu X.H., Li Q.L. (2016). Classification of Black Beans Using Visible and Near Infrared Hyperspectral Imaging. Int. J. Food Prop..

[153] Sun J., Lu X.Z., Mao H.P., Jin X.M., Wu X.H. (2017). A Method For Rapid Identification of Rice Origin by Hyperspectral Imaging Technology. J. Food Process Eng..

[154] Tian Y., Sun J., Zhou X., Wu X.H., Lu B., Dai C.X. (2020). Research on apple origin classification based on variable iterative space shrinkage approach with stepwise regression-support vector machine algorithm and visible-near infrared hyperspectral imaging. J. Food Process Eng..

[155] Zhu J.S., Cai J.R., Sun B.S., Xu Y.J., Lu F., Ma H.L. (2023). Inspection and classification of wheat quality using image processing. Qual. Assur. Saf. Crop Foods.

[156] Cong S.L., Sun J., Zhang B., Shi L., Zhou X., Wu X.H. (2025). A new method to identify the adulteration levels of Lonicerae Flos in Lonicerae Japonicae Flos using fluorescence hyperspectral imaging combined with optimized convolutional neural network. Microchem. J..

[157] Shi J., Liu C., Wu S., Huang X., Li Z., Zou X. (2020). Rapid Quantitative Characterization of Water Distribution Uniformity of Noodle Dough Sheet. Food Sci..

[158] Zhou X., Sun J., Mao H.P., Wu X.H., Zhang X.D., Yang N. (2018). Visualization research of moisture content in leaf lettuce leaves based on WT-PLSR and hyperspectral imaging technology. J. Food Process Eng..

[159] Shi L., Sun J., Zhang B., Wu Z.Q., Jia Y.L., Yao K.S., Zhou X. (2024). Simultaneous detection for storage condition and storage time of yellow peach under different storage conditions using hyperspectral imaging with multi-target characteristic selection and multi-task model. J. Food Compos. Anal..

[160] Sun J., Wu M.M., Hang Y.Y., Lu B., Wu X.H., Chen Q.S. (2019). Estimating cadmium content in lettuce leaves based on deep brief network and hyperspectral imaging technology. J. Food Process Eng..

[161] Zhang L., Sun J., Zhou X., Nirere A., Wu X.H., Dai R.M. (2020). Classification detection of saccharin jujube based on hyperspectral imaging technology. J. Food Process Preserv..

[162] Zhou X., Jun S., Yan T., Bing L., Hang Y.Y., Quansheng C. (2020). Hyperspectral technique combined with deep learning algorithm for detection of compound heavy metals in lettuce. Food Chem..

[163] Zhou X., Zhao C.J., Sun J., Cao Y., Yao K.S., Xu M. (2023). A deep learning method for predicting lead content in oilseed rape leaves using fluorescence hyperspectral imaging. Food Chem..

[164] Wang Z.Z., Li T.G., Du R., Yang N., Ping J.F. (2025). A high-efficiency lettuce quality detection system based on FPGA. Comput. Electron. Agric..

[165] Tian Y., Sun J., Zhou X., Yao K.S., Tang N.Q. (2022). Detection of soluble solid content in apples based on hyperspectral technology combined with deep learning algorithm. J. Food Process Preserv..

[166] Xu M., Sun J., Cheng J.H., Yao K.N., Wu X.H., Zhou X. (2023). Non-destructive prediction of total soluble solids and titratable acidity in Kyoho grape using hyperspectral imaging and deep learning algorithm. Int. J. Food Sci. Technol..

[167] Zheng X., Li Y., Wei W., Peng Y. (2019). Detection of adulteration with duck meat in minced lamb meat by using visible near-infrared hyperspectral imaging. Meat Sci..

[168] Crichton S., Kirchner S., Porley V., Retz S., von Gersdorff G.J.E., Hensel O., Weygandt M., Sturm B.J.M.s. (2017). Classification of organic beef freshness using VNIR hyperspectral imaging. Meat Sci..

[169] Shi Y., Wang Y.Y., Hu X.T., Li Z.H., Huang X.W., Liang J., Zhang X.A., Zheng K.Y., Zou X.B., Shi J.Y. (2023). Nondestructive discrimination of analogous density foreign matter inside soy protein meat semi-finished products based on transmission hyperspectral imaging. Food Chem..

[170] Sun J., Tang K., Wu X.H., Dai C.X., Chen Y., Shen J.F. (2018). Nondestructive identification of green tea varieties based on hyperspectral imaging technology. J. Food Process Eng..

[171] Tang N.Q., Sun J., Yao K.S., Zhou X., Tian Y., Cao Y., Nirere A. (2021). Identification of *Lycium barbarum* varieties based on hyperspectral imaging technique and competitive adaptive reweighted sampling-whale optimization algorithm-support vector machine. J. Food Process Eng..

[172] Yang N., Yuan M.F., Wang P., Zhang R.B., Sun J., Mao H.P. (2019). Tea diseases detection based on fast infrared thermal image processing technology. J. Sci. Food Agric..

[173] Cao Y., Li H.R., Sun J., Zhou X., Yao K.S., Nirere A. (2020). Nondestructive determination of the total mold colony count in green tea by hyperspectral imaging technology. J. Food Process Eng..

[174] Li L.Q., Xie S.M., Ning J.M., Chen Q.S., Zhang Z.Z. (2019). Evaluating green tea quality based on multisensor data fusion combining hyperspectral imaging and olfactory visualization systems. J. Sci. Food Agric..

[175] Zheng P.F., Adade S., Rong Y.N., Zhao S.G., Han Z., Gong Y.T., Chen X.Y., Yu J.H., Huang C.C., Lin H. (2024). Online System for Monitoring the Degree of Fermentation of Oolong Tea Using Integrated Visible-Near-Infrared Spectroscopy and Image-Processing Technologies. Foods.

[176] Xin X., Sun J., Shi L., Yao K.S., Zhang B. (2025). Application of hyperspectral imaging technology combined with ECA-MobileNetV3 in identifying different processing methods of Yunnan coffee beans. J. Food Compos. Anal..

[177] Tang N.Q., Jun S., Min X., Yao K.S., Yan C., Liu D.J. (2022). Identification of fumigated and dyed *Lycium barbarum* by hyperspectral imaging technology. J. Food Process Eng..

[178] You J., Li D.S., Wang Z., Chen Q.S., Ouyang Q. (2024). Prediction and visualization of moisture content in Tencha drying processes by computer vision and deep learning. J. Sci. Food Agric..

